# A Rare Occurrence of Stargardt Disease in a Quadragenarian Adult

**DOI:** 10.7759/cureus.30859

**Published:** 2022-10-29

**Authors:** Saket Y Maheshwari, Swarupa Chakole

**Affiliations:** 1 Ophthalmology, Jawaharlal Nehru Medical College, Datta Meghe Institute of Medical Sciences, Wardha, IND; 2 Community Medicine, Jawaharlal Nehru Medical College, Datta Meghe Institute of Medical Sciences, Wardha, IND

**Keywords:** genetic mutation, hereditary ailment, macular dystrophy, adult-onset, stargardt's disease

## Abstract

The retina is the light-sensitive layer of the human eye. The macula forms the central part of the retina. The character of light responsiveness is attributed to the presence of photoreceptor cells here. Stargardt's disease is the most common cause of hereditary macular dystrophy. It is linked to disease-causing sequence variations/mutations in the ABCA4 gene on chromosome 1p21-p13, which destroys rod and cone cells within the retina. The disc membranes of rod and cone outer segments include an ATP-binding cassette transport protein encoded by the ABCA4 gene. All trans-retinal conjugates are transported across disc membranes by the ABCA4 protein. Abnormally high amounts of lipofuscin pigments build up in the retinal pigment epithelium (RPE) due to mutations in the ABCA4 gene, leading to RPE cell loss and secondary photoreceptor cell degeneration. As a result of this disease, the central or detailed vision becomes blurred, and the patient may find it challenging to discern colours. The retina presents with a distinctive “beaten-bronze” appearance due to the presence of prominent yellow dots. The phenotypic form of Stargardt disease, known as fundus flavimaculatus, is characterized by the widespread distribution of flecks throughout the fundus, including the periphery. In the given case report, we present a 46-year-old male patient who presented with complaints of persistence of a blind spot in central vision, difficulty in identifying faces, distortion of letters while reading, decreased visual acuity and difficulty in adapting from light to dark settings as symptoms. The stepwise assessment of the patient led to the diagnosis of Stargardt's disease. The case report reflects the disease history, pathogenesis, manifestations, prognosis, differential diagnosis and treatment options for this rare presentation.

## Introduction

The most common juvenile macular degeneration (JMD) of the autosomal recessive (AR) type is known as Stargardt's disease [1]. Although it is classified as JMD, it does not necessarily mean that the onset of this disease has to begin at a young age (as in the given patient's case). Being AR, this problem manifests when both parents also possess a mutation of this gene. Under Stargardt, yellowish fatty flakes of pisciform or fishtail appearance get settled at close range of the macula at the level of retinal pigment epithelium (RPE) [2]. Most people with this rare disorder do not get completely blind, as peripheral vision is usually retained. Their centre vision may be impaired, making tasks like reading newspapers and face recognition challenging [3]. Generally, early onset is linked to a worse prognosis, even though the age of onset and illness progression vary greatly depending on the combination of particular disease-causing genes and modifiers. While null alleles are typically linked to more severe, earlier-onset disease, missense variants are generally associated with relatively mild, later-onset disease. However, some missense variants can also have significant functional effects comparable to nulls. A complex of p.Leu541Pro and p.Ala1038Val is an example. On the contrary, a milder phenotype is linked to late onset in life's journey [4]. The formation of fatty flakes is attributed to a substance called lipofuscin. The primary pathologic abnormality is the buildup of toxic lipofuscin pigments like N-retinylidene-N-retinylethanolamine (A2E) in the retinal pigment epithelial cells in Stargardt's disease. This buildup causes photoreceptor destruction and significant visual loss [5].

## Case presentation

A 46-year-old male patient presented to an ophthalmology clinic with chief complaints of blurring and diminution of vision (for distance as well as near), a blank patch occurring in the central vision, and distortion of letters while reading any printed text for the past two years. He reported a progressive and gradual loss in the ability of his eyes to distinguish between faces while viewing from a distance. The patient reported a prescription of spectacles for the last eight years and added that he had no history of any head or ocular trauma in the past. There was no significant family history of visual problems. The patient's father had hypertension, and his mother was diagnosed with diabetes mellitus. He abstained from all sorts of addictions like chewing tobacco, smoking and consumption of alcohol. He was a civil engineer by profession. He was well-nourished and did not suffer from any chronic illness.

**Figure 1:**
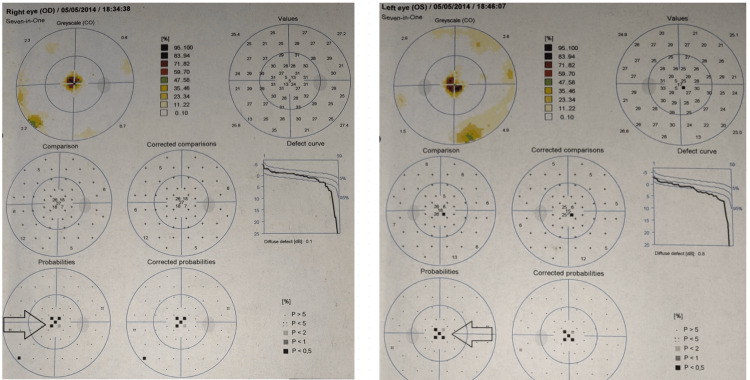
Perimetry examination of right and left eye.

**Figure 2:**
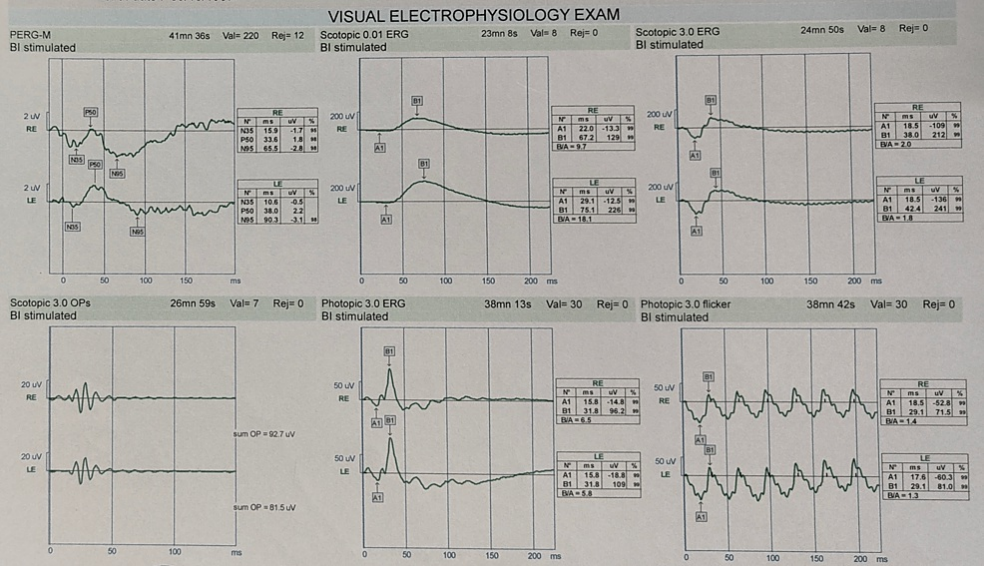
Visual electrophysiology exam of the patient.

His systemic and general examination was within normal limits. Upon initial ocular examination with the Snellen chart, the patient had a dynamic visual acuity (DVA) of 6/24 in the right eye without spectacle correction and 6/12 after refractive correction, whereas the left eye presented with a refractive power of 6/9 before correction and 6/7.5 after correction. Amsler grid assessment showed metamorphopsia. The Ishihara test represented that both eyes could only appreciate demonstration plates. Applanation tonometry revealed an intraocular pressure of 12 mm of Hg in both the right and the left eye. A slit lamp examination revealed the pupil of the left eye to be round, regular and reacting with no relative afferent pupillary defect (RAPD). In contrast, the pupil of the right eye was diagnosed with RAPD. Perimetry examination demonstrated the presence of a central scotoma and a reduction in the foveal threshold in both eyes, as shown in Figure 1. Upon optic disc examination, the cup/disc ratio was 0.3. Electroretinogram (ERG) test was done to find abnormalities in retinal functioning (Figure 2). It suggested subnormal scotopic and normal photopic response of retinal functioning.

**Figure 3:**
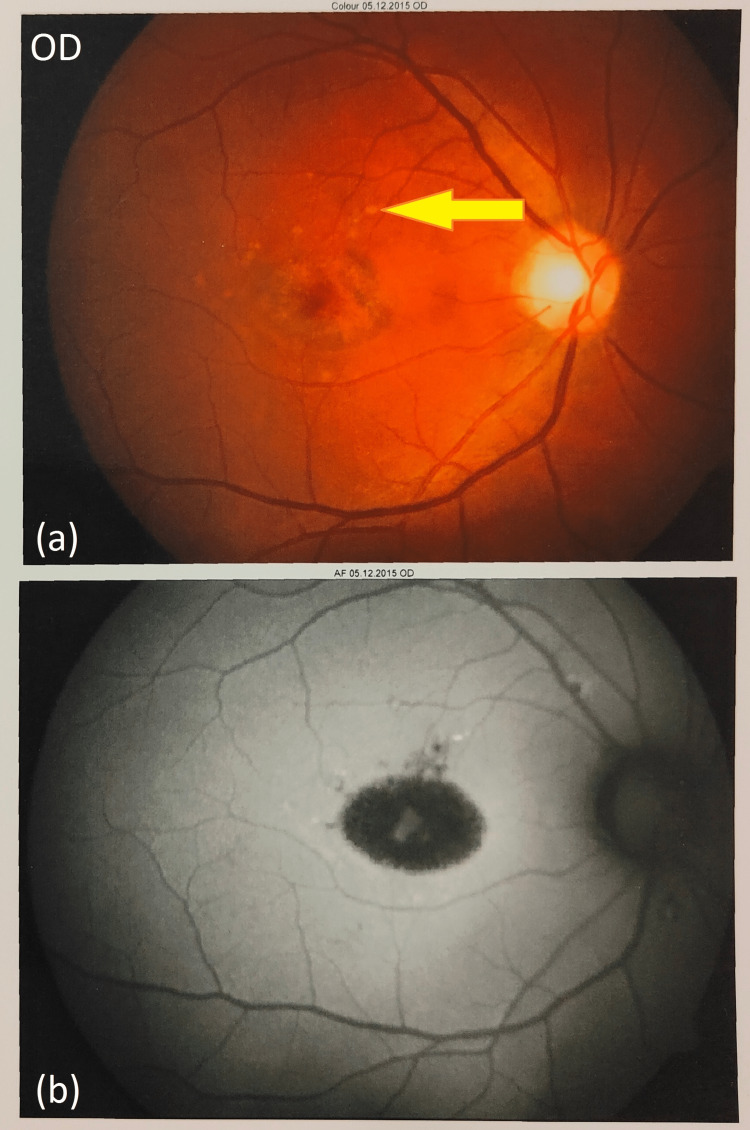
Colour fundus photograph and fundus autofluorescence tests (OD). (a) The colour fundus photograph of the right eye shows yellow pisciform flecks formed due to macular atrophy. (b) The fundus autofluorescence test of the right eye. Bull's eye maculopathy is seen due to central macular hypo autofluorescence surrounded by a hyper autofluorescence ring. Hypo autofluorescence is observed due to decreased metabolic activity within the RPE. OD: Oculus dexter

**Figure 4:**
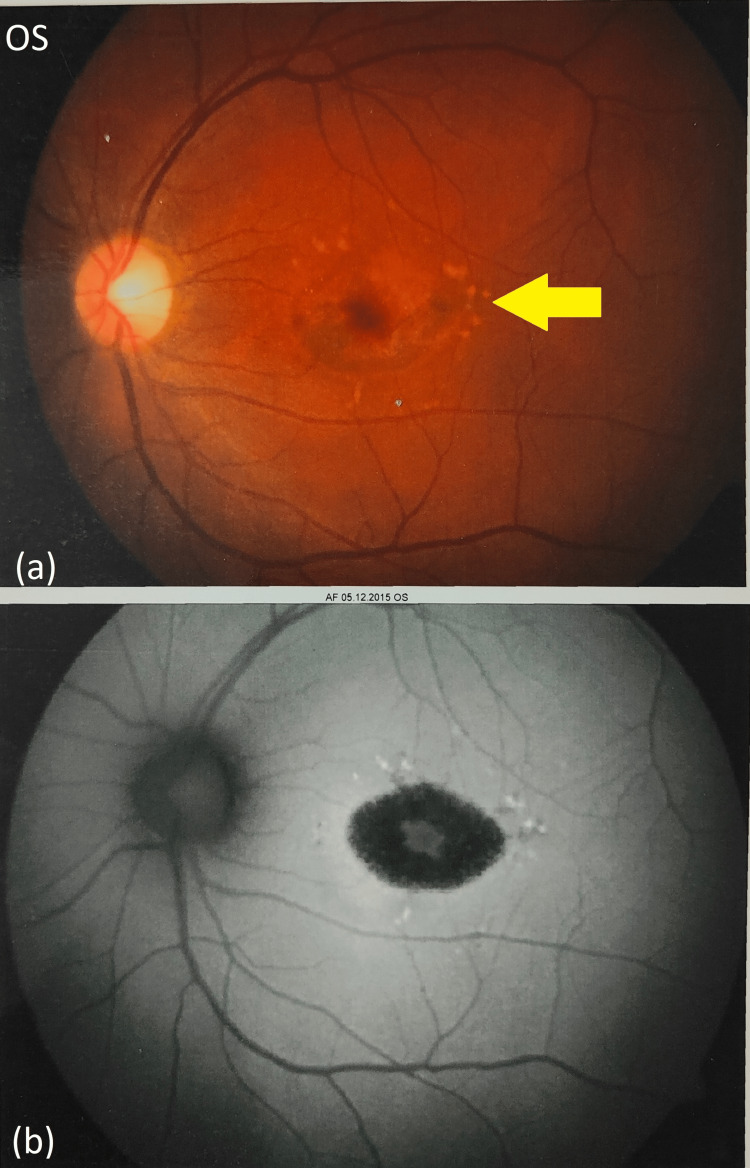
Colour fundus photograph and fundus autofluorescence tests (OS). (a) Colour fundus photograph of the left eye shows yellow-white flecks formed due to macular atrophy. (b) FA of the left eye shows bull's eye maculopathy. OS: Oculus sinister

Detailed retinal investigations included colour fundus photography (CFP) and fundus autofluorescence (FA) of the right eye (Figures 3a, 3b) and left eye (Figures 4a, 4b). CFP revealed a characteristic “beaten bronze appearance” of the retina formed due to surrounding yellow-white flecks extending outward from the fovea. FA indicated classic bull's eye maculopathy. Fluorescein angiography demonstrated a hallmark “silent/dark choroid” appearance developed due to the unnatural obscuring of choroidal fluorescence.

**Figure 5:**
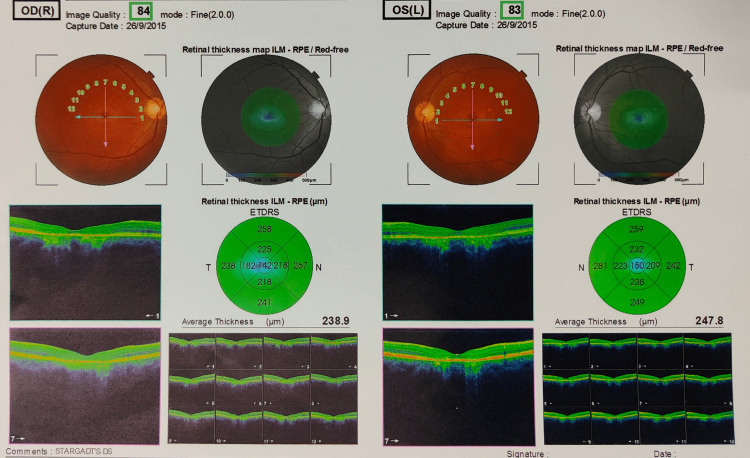
3D OCT (optical coherence tomography) of the patient. The OCT scan depicts a retinal thickness of 238.9 μm in the right eye and 247.8 μm in the left eye. Gross central foveal thinning can be visualised with the atrophy of the outer retinal and inner choroidal layers.

For confirmation of the diagnosis, 3D OCT (optical coherence tomography) was chosen as the desired test of choice (Figure 5). The patient had a central foveal thickness of 142 μm OD and 150 μm OS. Therefore, OCT was indicative of foveal thinning due to retinal atrophy. It depicted thinning and disorganization of the inner segment-outer segment (IS-OS) junction of the photoreceptors in the macula.

**Figure 6:**
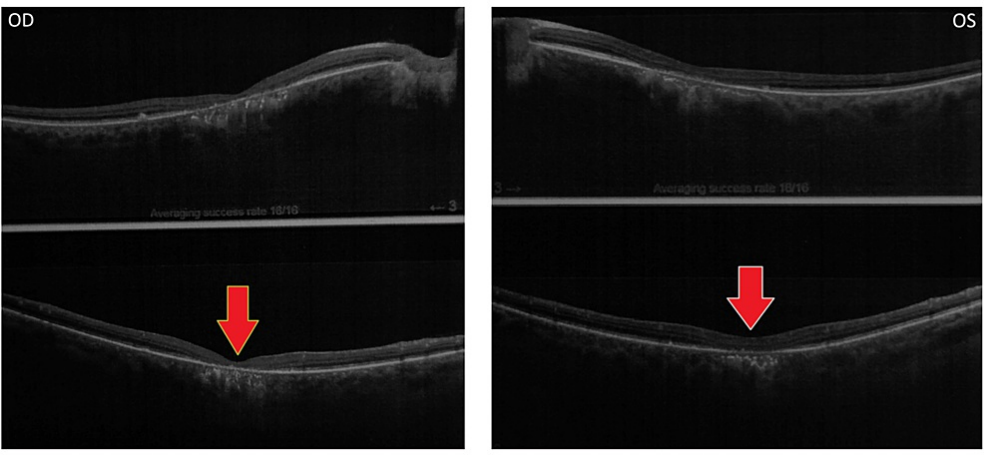
Comparison of extent of the foveal thinning. The given image compares the extent of foveal thinning in the right and left eye.

Foveal thinning and blurring of vision were more prominent in the retina of the right eye as compared to the left eye (Figure 6).

The reports were suggestive of Stargardt's disease. After the diagnosis, the patient was counselled regarding the prognosis of his condition. He was suggested to revisit the hospital for a follow-up after one year or in case of an emergency.

## Discussion

A German ophthalmologist named Karl Bruno Stargardt pioneered the discovery of Stargardt's disease in 1909 [6]. Mutations in the ABCA4 gene are considered the most common aetiology for the occurrence of Stargardt's disease. An autosomal recessive pattern of disease inheritance is observed when a genetic mutation occurs within the ABCA4 gene. Sometimes, this condition is inherited in an autosomal dominant pattern when the disease is caused due to ELOV4 gene mutation [1]. Vitamin A is considered a crucial element for the normal functioning of the human eye as it is essential for the production of opsins. The disc membranes of rod and cone outer segments include an ATP-binding cassette transport protein encoded by the ABCA4 gene [7]. This transport protein is involved in the transmembrane transportation of vitamin A intermediates from photoreceptors to RPE. Hence, this gene impacts the way one's eyes utilize vitamin A as it has a significant role in hydrolyzing N-retinylidene-phosphatidylethanolamine (N-ret-PE) to all-trans-retinal and phosphatidylethanolamine [8]. The inefficient hydrolysis of N-ret-PE due to ABCA4 dysfunction leads to the accumulation of lipofuscin and toxic bisretinoid compounds like di-retinoid-pyridinium-ethanolamine within the photoreceptor membranes [9]. Vitamin A by-products or dimers accumulate in the RPE after phagocytosis of the outer photoreceptor segments. Excessive lipofuscin accumulation leads to this fatty substance's aggregation on the macula in yellowish aggregates. The fatty substance gradually kills the cells that are sensitive to light, impairing one's central vision [10]. Post this, the lipofuscin-affected macula leads to bilateral progressive dysfunctioning of the eye. Driving a car or riding a bike is not recommended for such people as the point where a person tries to concentrate through their eyes appears obscure. Although errors in the ABCA4 gene most frequently cause Stargardt, some patients may have errors in the ELOVL4, PROM1 and RDS genes (also known as PRPH2) [11]. Based on ancillary testing and clinical examination findings, Fishman described four stages of Stargardt's disease. Stage one comprises a ring of pisciform-shaped flecks within one fovea disc diameter (DD). Stage two is constituted of pisciform flecks beyond one DD from the margin of the fovea. Stage three indicated Chorio-capillary atrophy along with subnormal cone and rod amplitudes under ERG, whereas stage four showed diffusely resorbed flecks and extensive RPE atrophy throughout the entire fundus [12].

Bull's eye macular lesions are seen in age-related macular degeneration (ARMD), Stargardt disease, chloroquine retinopathy (CR) and cone dystrophy (CD) [13]. The possibility of CR was ruled out as the patient had no supporting medical history of chloroquine drug intake. The ERG assessment revealed that the patient had a subnormal scotopic and normal photopic response of retinal function. This interpretation helped the doctor to exclude the likelihood of cone dystrophy in the patient as CD demonstrates a substantial loss of cone functions with normal rod responses. Further, the possibility of ARMD was rejected due to several reasons. ARMD is generally seen in the senile population above 60 years of age. In severe cases of dry ARMD, CFP reveals prominent choroidal vessels due to the atrophy of inner choroidal and outer retinal layers. This presentation was not seen in the CFP of the given patient. Moreover, the drusens seen in cases of ARMD are circular and distributed bilaterally in a less symmetrical fashion. This observation was again dismissed in the CFP of the patient.

For the treatment of Stargardt's disease, there are some principal avenues of intervention being explored. Emixustat hydrochloride, when taken orally, acts as a visual cycle modulator as it inhibits retinoid isomerohydrolase. This reduces the conversion of all-trans-retinyl ester to 11-cis-retinol and prevents the accumulation of A2E, a toxic byproduct of the visual cycle. Complement inhibition by a C5 inhibitor drug called avacincaptad pegol may prevent the formation of membrane attack complex, consequently reducing cell death caused by the destruction of the cell membrane. This drug is delivered by intravitreal injection and is explored as a potential treatment option for Stargardt's disease (STGD). Daily intake of C20-D3-vitamin A molecule ALK-001 by the oral route is assessed as a viable treatment option for STGD as it impedes vitamin A dimerization. The concept of injecting a normally functioning ABCA4 gene as a substitute for the mutated one has recently gained wide attention from scientists. This possibility could be surveyed using equine infectious anaemia lentivirus (EIAV) for gene transfer. Lastly, stem cell therapy is being attempted by producing a two-layered patch covered by a thin plastic film. According to the proposed model, one layer contains the precursors to rods and cones, while the other layer consists of mature retinal pigment epithelial cells [14].

 Since the treatment modalities are still under comprehensive assessment, the doctor suggested some low vision aids like magnifiers and prisms for improving central vision. While counselling, he asked the patient to abstain from excess dietary intake of vitamin A as it might worsen the condition. He also stated some basic facts about handling the spectacles to the patient. The points of advice included: Periodically get the spectacles adjusted according to the texture of the face. Rinse the specs under running tap water and wipe them dry using dust-free, link-free soft material. Do not use paper towels, silicone tissues, facial tissues, or old rags with embedded dirt that may scratch the lenses. Use only a microfibre cloth to wipe the spectacle lens. Clean the nose pads and hinges using a soft toothbrush. Never leave spectacles on a car's dashboard or near any heat source, as this may stretch the edge and cause the lenses to crack or craze due to excessive heat and extreme temperature variation. If the patient is allergic to metal frames, they should use only hypo-allergic metal frames (e.g., titanium).

## Conclusions

In the given case, a male patient presented to the hospital with chief complaints of blurring of vision, distortion of letters while reading, and difficulty in identifying faces. Various investigations like the Snellen chart and Amsler grid assessment supported his claim of loss of visual acuity, whereas detailed investigations like fundus fluorescein angiography (FFA), FA and optical coherence tomography pointed towards the diagnosis of Stargardt's disease. The chosen case is distinctive as Stargardt's disease is considered a rare ailment.

Although this disease is tagged as the most common cause of JMD, the given case describes the manifestation of it in a patient of 46 years of age. Unfortunately, no specific treatment or surgery to cure Stargardt's disease has been devised until now. Hence, robust research in pharmacological interventions to treat retinal pathologies is the need of the hour. In the present times, the best possible way to control this disease progression can be by using appropriate low-vision aids and spectacles for the patient. Visual rehabilitation and proper eyecare routine are helpful too.

## References

[1] (2022). Stargardt macular degeneration: MedlinePlus Genetics. https://medlineplus.gov/genetics/condition/stargardt-macular-degeneration/..

[2] Armstrong JD, Meyer D, Xu S, Elfervig JL (1998). Long-term follow-up of Stargardt’s disease and fundus flavimaculatus. Ophthalmology.

[3] (2022). Stargardt disease, RNIB. https://www.rnib.org.uk/your-eyes/eye-conditions-az/stargardt-disease/.

[4] (2022). Clinical and molecular characteristics of childhood-onset Stargardt disease. https://pubmed.ncbi.nlm.nih.gov/25312043/.

[5] Radu RA, Mata NL, Nusinowitz S, Liu X, Sieving PA, Travis GH (2003). Treatment with isotretinoin inhibits lipofuscin accumulation in a mouse model of recessive Stargardt's macular degeneration. Proc Natl Acad Sci U S A.

[6] (2022). Stargardt disease, hereditary ocular diseases. https://disorders.eyes.arizona.edu/handouts/stargardt-disease.

[7] (2022). ABCA4 - an overview. https://www.sciencedirect.com/topics/biochemistry-genetics-and-molecular-biology/abca4.

[8] (2022). Stargardt disease. https://www.nei.nih.gov/learn-about-eye-health/eye-conditions-and-diseases/stargardt-disease.

[9] Lindner M, Lambertus S, Mauschitz MM (2017). Differential disease progression in atrophic age-related macular degeneration and late-onset Stargardt disease. Invest Ophthalmol Vis Sci.

[10] (2022). Genetic characterization of Stargardt clinical phenotype in South Indian patients using sanger and targeted sequencing. https://pubmed.ncbi.nlm.nih.gov/31934596/.

[11] (2022). Stargardt macular dystrophy - causes, symptoms and treatments. https://www.fightforsight.org.uk/about-the-eye/a-z-eye-conditions/stargardt-macular-dystrophy/.

[12] (2022). Diagnosis and management of Stargardt disease. https://www.aao.org/eyenet/article/diagnosis-management-of-stargardt-disease.

[13] (2022). Seeking therapies for Stargardt macular dystrophy. https://retinatoday.com/articles/2019-july-aug/seeking-therapies-for-stargardt-macular-dystrophy.

[14] (2022). Stargardt disease research advances — Foundation Fighting Blindness. https://www.fightingblindness.org/research/stargardt-disease-research-advances-6.

